# Upper airway obstruction due to a change in altitude: first report in fifty years

**DOI:** 10.1186/s40463-016-0121-y

**Published:** 2016-02-01

**Authors:** Oleksandr Butskiy, Donald W. Anderson

**Affiliations:** Division of Otolaryngology Head and Neck Surgery, Department of Surgery, Vancouver General Hospital & University of British Columbia, Vancouver, BC Canada; Gordon & Leslie Diamond Health Care Centre, 4th. Fl. 4299B-2775 Laurel Street, Vancouver, BC V5Z 1 M9 Canada

**Keywords:** Airway obstruction, Air travel, Neck mass, Laryngocele

## Abstract

**Background:**

Air travel mostly causes minor ear, nose and throat complaints. We describe a second report in literature of airway obstruction caused by a drop in atmospheric pressure during a routine commercial flight.

**Case presentation:**

A 54-year-old male was referred to a head and neck surgeon with a 2 cm left submandibular mass that would enlarge during commercial flights. As the plane gained elevation, the mass would grow and cause him to become stridorous and short of breath. The shortness of breath and stridor would only resolve upon landing of the plane. A CT scan showed a large air sac extending from the larynx at the level of the true vocal cords up to the angle of the mandible. Based on the history and the CT findings a diagnosis of a laryngocele was made. The laryngocele was excised using an external approach, resolving the patient’s difficulty with flying.

**Conclusion:**

This article reports a rare case of upper airway obstruction caused by atmospheric pressure changes during air travel. The reported case is of significance as only a few uncomplicated laryngoceles have been reported to cause airway distress in the literature. This report highlights the epidemiology, presentation, complication and management of laryngoceles.

## Background

With the exception of otic barotrauma, air travel has only been reported to cause minor complaints in the ear, nose and throat [1]. We describe a case of upper airway obstruction during a routine commercial flight. Based on our search of Embase®, Pubmed, Google Scholar, and Web of Science™ databases (last search June 2015), we believe this is to be the second case report of airway obstruction caused by airplane’s change in altitude [2].

## Case presentation

**Fig. 1:**
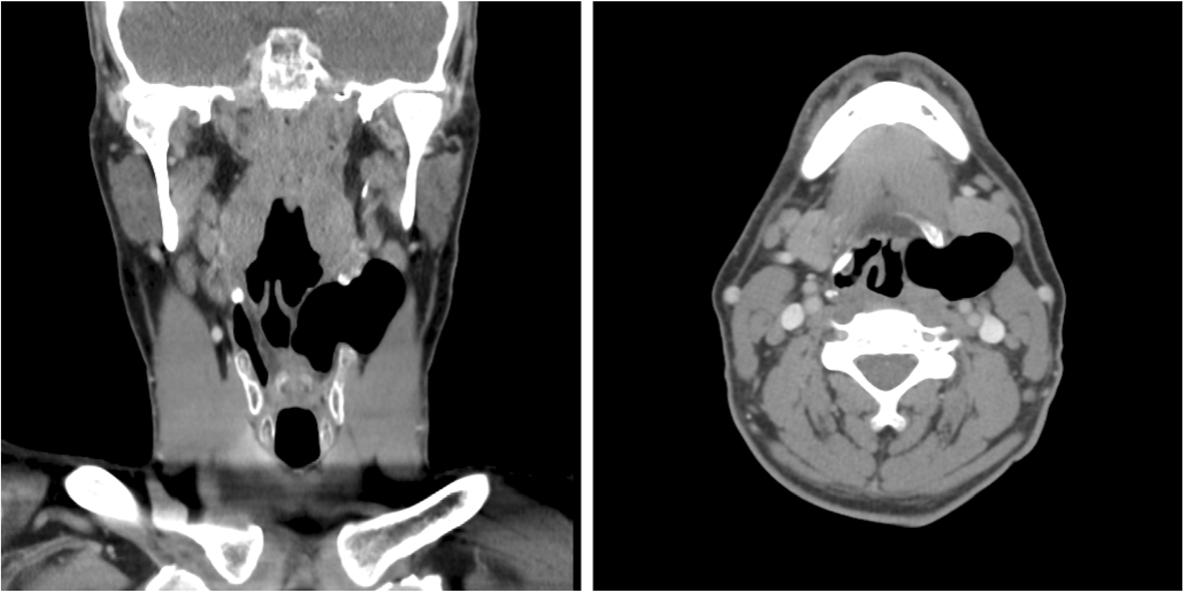
CT of the neck with contrast demonstrating a laryngocele

A 54-year-old male smoker was referred to a head and neck surgeon with a 2 cm left submandibular mass. On history, the patient described a chronic non-painful left neck mass that fluctuated in size over the years. The patient’s chief complaint, however, was the problem he experienced during commercial flights. During the plane’s ascent, the left neck mass would enlarge, and he would become short of breath and stridorous. These symptoms would only resolve upon the plane’s descent. During these episodes he never sought medical attention. However, these episodes were severe enough that he has been avoiding all air travel, and he only pursued surgical consultation to attend his daughter’s wedding abroad. On palpation of the neck, no neck mass, swelling, nor lymphadenopathy were appreciated. Flexible laryngoscopy showed an infantile type epiglottis. A CT scan of the neck was ordered and showed a large air-containing sac in the left neck, extending from the level of the vocal cords to the level of the angle of the mandible. The air sac, insinuated between the left strap muscles and left sternocleidomastoid, was causing mass effect on the left submandibular gland and the laryngeal structures (Fig. 1). Based on the history and CT findings, a diagnosis of a laryngocele with internal and external components was made and the patient was counseled regarding its surgical excision.

### Discussion and surgical management

A laryngocele is an air filled abnormal dilation of the laryngeal saccule communicating with the laryngeal lumen. The exact etiology of laryngoceles is unknown. Some authors attribute laryngoceles to congenitally present dilation of the saccule exacerbated by factors that increase intra-glottic pressure such as professional trumpet playing [3]. It is important to remember that laryngoceles are know to present in the setting of laryngeal malignancy, secondary to partial of complete obstruction of the saccular orifice [4]. Laryngoceles are rare. Traditionally, the incidence of laryngocele was reported to be approximately 1 in 2.5 million people [5]. The true incidence of laryngoceles is controversial, as more recent report suggest that laryngoceles might be more common than originally thought [6]. Two anatomical variations of laryngoceles have been reported: internal to the thyroid cartilage and a combined type, consisting of external and internal components. The authors of a recent review reported that the treatment of laryngoceles depends on the anatomical variation: internal laryngoceles tend to be treated with microlaryngoscopy with CO2 laser, while the combined laryngoceles tend to be excised through an external incision [7].

**Fig. 2:**
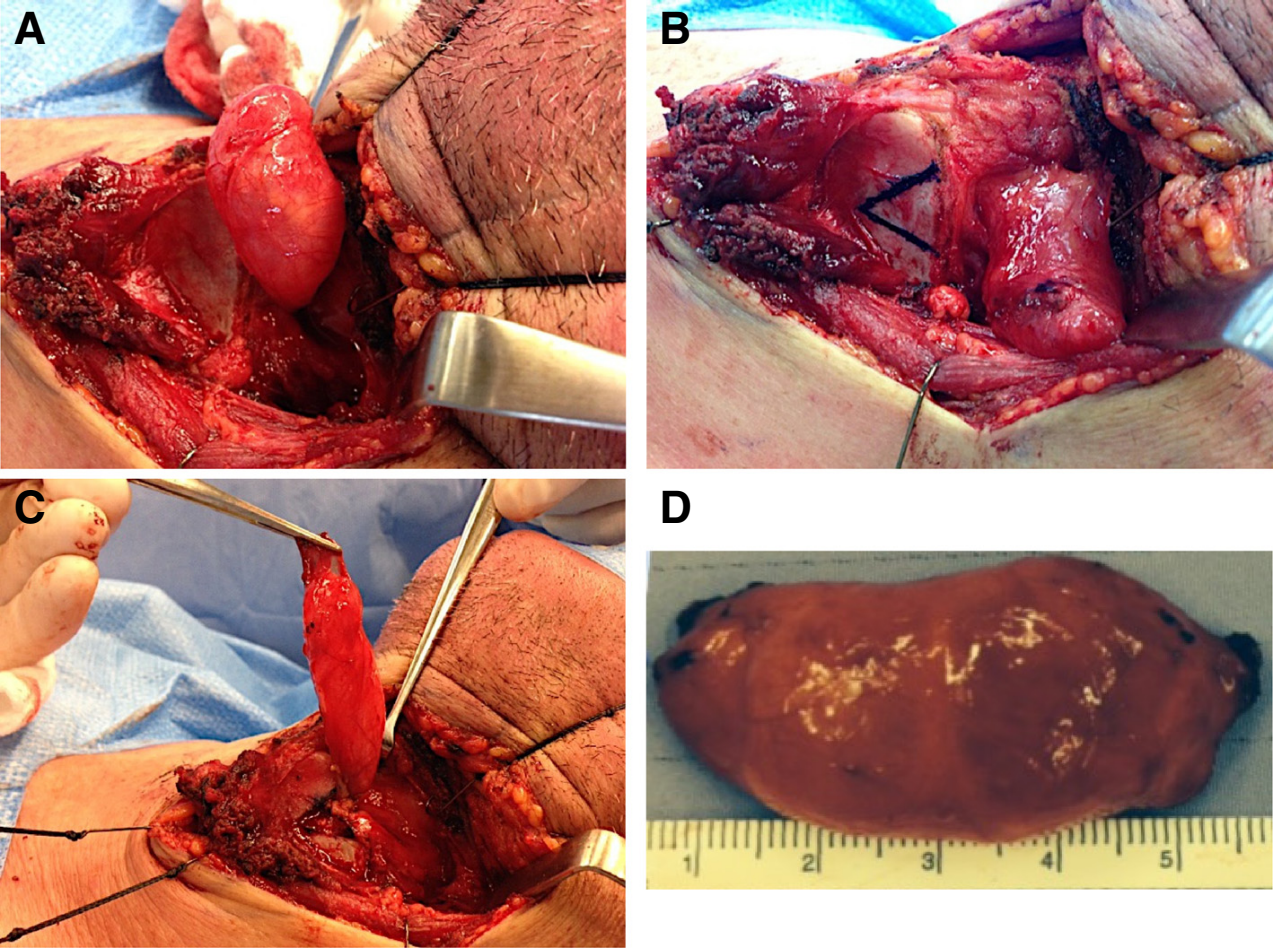
Resection of the laryngocele. (**a**) Strap muscles reflected inferiorly, bringing the laryngocele into view; (**b**) Planning to resect a portion of left thyroid lamina to gain further exposure; (**c**) Laryngocele dissected away from the paraglottic space down to the laryngeal ventricle; (**d**) Laryngocele delivered out of the neck

Given the size of the external component of the laryngocele presented in this report, an external approach, to the laryngocele excision was taken. A detailed description of the external surgical approach is available elsewhere [8]. In brief, a lateral thyrotomy without tracheostomy was chosen to resect the laryngocele. Strap muscles were reflected down together with the raised left thyroid ala perichondrium (Fig. 2a). An inverted triangular section of the left thyroid lamina was resected, taking care to stay anterior and parallel to the left oblique line (Fig. 2b). The laryngocele was dissected away from the surrounding paraglottic space down to the laryngeal ventricle (Fig. 2c). The communication between the laryngocele and the laryngeal ventricle was then clamped, tied and cut, delivering the laryngocele out of the neck (Fig. 2d). Following wound closure, the patient was successfully extubated in the operating room. He spent the night in the hospital for observation, and was discharged home with no changes in his voice, swallowing or breathing.

The patient’s follow up consisted of one office visit, 2 weeks after the operation, and one and a half year phone follow up. He was able to return to work 9 days after the operation and had no complaints at any time. He resumed air travel 3 months following his surgery, and he has not experienced airway obstruction or neck swelling during flights again.

The presented case highlights a typical patient who might present with a laryngocele: a male in his fifth or sixth decade referred with a non-tender neck mass that fluctuates in size [7]. The unusual part of the presented case is the airway obstruction caused by the laryngocele during air travel. Uncomplicated laryngoceles rarely cause airway obstruction [3]. Infected laryngoceles, or laryngopyoceles, can on occasion lead to airway distress [7] and can potentially be lethal [9]. The airway obstruction experienced by the patient presented in this case was likely due to the drop in the atmospheric pressure in the cabin of an airplane. If the junction of the laryngocele with the laryngeal saccule was intermittently obstructed, the drop in air pressure during the plane’s ascent would have led to laryngocele expansion, explaining the patient’s symptoms.

We searched Embase®, Pubmed, Google Scholar, and Web of Science™ databases (last search June 2015) and found one case reports from 50 years ago of airway obstruction during air travel caused by a laryngocele [2]. In addition, we also found a more recent brief communication by an ophthalmologist recounting her experiences from a commercial flight. Twenty minutes into a flight, she was asked to assist a passenger experiencing bulging on the side of the neck. It is unclear if the passenger had symptoms of airway obstruction. This bulge resolved as the plane made an emergency landing. The author of this brief communication did not follow the patient into the hospital, and was writing to request an opinion with regard to what might have caused this unusual presentation [10]. Given the similarities to the presented case, it is likely that the passenger might have had reversible airway obstruction due to a laryngocele.

## Conclusions

The presented case is the second case report of upper airway obstruction during air travel. Given the ubiquity of air travel, it is likely that other patients with laryngoceles have experienced at least some worsening of their symptoms during airplane’s ascent. We encourage practitioners to question the rare patient that presents with a suspicion of a laryngocele about symptom changes with air travel. As illustrated in this case, a change in symptoms during the ascent and descent of air travel can potentially support the physician’s diagnostic suspicion of a laryngocele.

## Consent to publish

Patient provided written informed consent for publication of the case report. Editor-in-chief was provided with a copy of the written consent.
